# Investigating Natural Product Inhibitors of IKKα: Insights from Integrative In Silico and Experimental Validation

**DOI:** 10.3390/molecules30092025

**Published:** 2025-05-02

**Authors:** Muhammad Yasir, Jinyoung Park, Eun-Taek Han, Jin-Hee Han, Won Sun Park, Jongseon Choe, Wanjoo Chun

**Affiliations:** 1 Department of Pharmacology, Kangwon National University School of Medicine, Chuncheon 24341, Republic of Korea; yasir.khokhar1999@gmail.com (M.Y.); jinyoung0326@kangwon.ac.kr (J.P.); 2 Department of Medical Environmental Biology and Tropical Medicine, Kangwon National University School of Medicine, Chuncheon 24341, Republic of Korea; ethan@kangwon.ac.kr (E.-T.H.); han.han@kangwon.ac.kr (J.-H.H.); 3 Department of Physiology, Kangwon National University School of Medicine, Chuncheon 24341, Republic of Korea; parkws@kangwon.ac.kr; 4 Department of Microbiology and Immunology, Kangwon National University School of Medicine, Chuncheon 24341, Republic of Korea; jchoe@kangwon.ac.kr

**Keywords:** IKKα, IκBα, NF-κB, inflammation, pharmacophore modeling, molecular docking, molecular dynamic simulation, RAW 264.7 cells

## Abstract

Nuclear factor-κB (NF-κB) signaling plays a pivotal role in regulating immune responses and is strongly implicated in cancer progression and inflammation-related diseases. The inhibitory κB kinases (IKKs), particularly IKKα, are central to modulating NF-κB activity, with distinct roles in the canonical and non-canonical signaling pathways. This study investigates the potential of selectively targeting IKKα to develop novel therapeutic strategies. A receptor–ligand interaction pharmacophore model was generated based on the co-crystallized structure of IKKα, incorporating six key features, two hydrogen bond acceptors, two hydrogen bond donors, one hydrophobic region, and one hydrophobic aromatic region. This model was used to virtually screen a diverse natural compound library of 5540 molecules, yielding 82 candidates that matched the essential pharmacophore features. Molecular docking and molecular dynamics simulations were subsequently employed to evaluate binding conformations, stability, and dynamic behavior of the top hits. The end-state free energy calculations (gmx_MMPBSA) further validated the interaction strength and stability of selected compounds. To experimentally confirm their inhibitory potential, key compounds were tested in LPS-stimulated RAW 264.7 cells, where they significantly reduced IκBα phosphorylation. These findings validate the integrative computational-experimental approach and identify promising natural compounds as selective IKKα inhibitors for further therapeutic development in cancer and inflammatory diseases.

## 1. Introduction

Nuclear factor-κB (NF-κB) transcription factors are vital regulators of both the innate and adaptive immune responses, and they also play a central role in the development and progression of cancer [1,2,3]. These factors contribute to several critical processes, such as enabling preneoplastic and malignant cells to evade apoptosis, promoting tumor angiogenesis, and invasiveness. Because of these functions, the signaling pathways that activate NF-κB complexes present promising targets for developing new chemotherapeutic strategies [2,4,5,6,7].

The activation of NF-κB is primarily regulated by inhibitory κB kinases (IKKs), which become overactive when cellular homeostasis is disrupted [8,9]. This overactivity is marked by increased constitutive activity of IKKα and IKKβ, leading to elevated NF-κB expression and its subsequent translocation into the nucleus [10]. IKKs function as upstream regulators of NF-κB activity by existing as either homo- or heterodimers, bound to inhibitory proteins called IκBs. In the canonical pathway, IKK activation leads to the phosphorylation, ubiquitination, and proteolytic degradation of IκBs, which allows NF-κB complexes to migrate into the nucleus and activate specific gene expression [11,12,13,14]. On the other hand, the non-canonical pathway involves the phosphorylation and processing of the NF-κB precursor protein p100, which then generates p52/RelB dimers that drive the expression of a distinct set of genes [15,16,17,18,19].

Research has shown that IKKα and IKKβ play distinct but essential roles in regulating global NF-κB signaling. IKKβ is primarily involved in the canonical pathway, where it activates p65 RelA–p50 heterodimers. Its inhibition has been shown to reduce pro-inflammatory gene expression in various cell types, which is crucial since many pro-inflammatory molecules contribute to cancer progression [15,20,21,22]. Although IKKα plays a less prominent role in the canonical pathway, it is crucial in the non-canonical pathway. IKKα catalyzes the phosphorylation and processing of p100, generating p52/RelB dimers, which activate a specific subset of genes [15,23].

In recent years, much attention has been given to selectively inhibiting IKKα. Over the last two decades, several IKKα inhibitors have been discovered, mainly for the treatment of inflammatory conditions [24,25,26,27,28]. However, recent findings suggest that inhibiting IKKβ can lead to significant toxic effects, including inflammatory skin diseases and increased sensitivity of the colonic epithelium to various types of damage [28,29,30]. In contrast, IKKα inhibition seems to offer a safer and more targeted approach. For example, a natural compound called noraristeromycin (NAM) has been found to selectively inhibit IKKα. NAM prevents the phosphorylation and degradation of IκBα after TNFα stimulation, and it also blocks the phosphorylation of the NF-κB subunit p65 [27,31,32]. These findings highlight the potential of NAM and other IKKα inhibitors as therapeutic agents.

**Figure 1:**
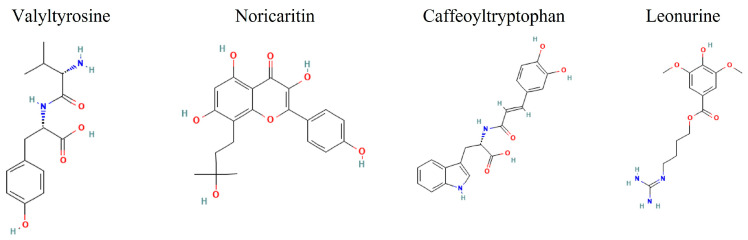
The 2D images of the screened candidate compounds against IKKα.

Given the growing body of evidence supporting the critical role of IKKα in cancer, particularly in prostate, breast, and pancreatic cancers, there is an urgent need for selective IKKα inhibitors [32,33]. These inhibitors would not only provide deeper insights into the role of IKKα in cancer development and progression but also offer a promising strategy for developing targeted therapies for cancers where IKKα is implicated in tumor growth and metastasis. However, natural products against IKKα have not been widely explored. Therefore, filling out the remaining gaps, this study aims to identify promising lead compounds for targeted IKKα inhibition from the pool of natural compounds, thus proposing Valyltyrosine, Noricaritin, Caffeoyltryptophan, and Leonurine as potent drug candidates (Figure 1).

## 2. Results and Discussion

### 2.1. Pharmacophore Modeling

One of the major challenges in drug discovery is addressing the side effects caused by the non-selective action of compounds, which can lead to unintended interactions with off-target proteins [34,35]. To mitigate this issue, pharmacophore modeling emerges as a powerful strategy. By focusing on the essential features required for selective binding, pharmacophore modeling enables the identification and screening of compounds that closely match the active binding pocket of a target protein, thereby improving specificity and reducing off-target effects [36,37,38].

Previously, pharmacophore studies for IKKβ had shown some promising results and a number of potential inhibitors were suggested against the IKKβ [39,40]. However, a recent study suggested that inhibiting IKKβ can lead to toxic effects such as inflammatory skin diseases and increased sensitivity of the colonic epithelium [28,29,30].

**Figure 2:**
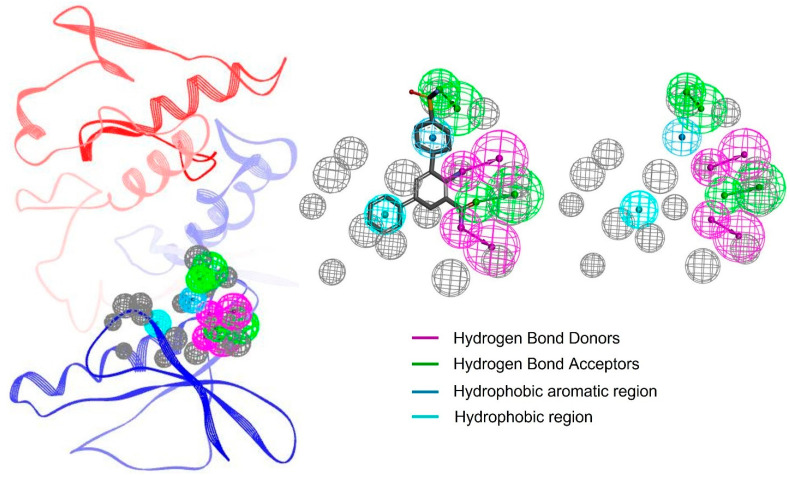
The pharmacophore features of the protein–ligand complex. Each feature is highlighted in a different color. The excluded volume areas are colored as grey.

The resulting pharmacophore model of this study for IKKα was critical for binding efficacy and selectivity. A total of 10 pharmacophore models were predicted by the RLIPG approach in Discovery Studio (Supplementary data Table S1); therefore, the top pharmacophore was chosen for further library screening. The model included two hydrogen-bond acceptors (green), two hydrogen-bond donors (magenta), a hydrophobic region (cyan), and a hydrophobic aromatic region (blue). These features correspond to functional groups on the ligand that can form strong and specific interactions with complementary regions of the target protein. Additionally, the model incorporated excluded volume areas (gray), representing sterically inaccessible regions that restrict the binding of non-specific compounds (Figure 2).

The hydrogen-bond acceptors and donors play a pivotal role in stabilizing the ligand within the binding pocket, forming strong electrostatic interactions with complementary residues on IKKα. These interactions are complemented by the hydrophobic region, which enhances binding affinity by interacting with nonpolar residues in the protein’s active site. The inclusion of excluded volume features ensures that only compounds with an optimal size and shape can align with the pharmacophore, further refining selectivity [30,41,42].

By integrating critical interaction features such as hydrogen bond donors and acceptors, hydrophobic regions, and excluded volumes, the pharmacophore model serves as a precise blueprint for identifying ligands with high binding specificity. This model not only facilitates efficient virtual screening but also enhances lead optimization by filtering out compounds unlikely to interact with IKKα’s active site. As a result, the selected hits are more likely to exhibit selective binding to IKKα while minimizing interactions with structurally similar kinases, thereby reducing the risk of off-target effects and potential toxicity. This strategic design approach plays a crucial role in improving the quality and therapeutic relevance of the identified inhibitors.

### 2.2. Molecular Docking

The CDocker module in Discovery Studio evaluates the binding affinity and interaction between small molecules and a target protein by calculating two key energy parameters: CDocker Energy and CDocker Interaction Energy. CDocker Energy (measured in kcal/mol) reflects the total internal and binding energy of the ligand–protein complex. A lower CDocker Energy value indicates a more stable and energetically favorable binding conformation, signifying stronger interactions, whereas the CDocker Interaction Energy (also in kcal/mol) measures the direct interaction strength between the ligand and the protein [43,44,45]. Highly negative interaction energy values suggest that the compound forms strong interactions with the active site residues of IKKα. Out of the 82 pharmacophore screened compounds, 22 compounds were excluded during the ligand preparation step by Discovery Studio’s default settings. The Discovery studio has built an exclusion criteria for the compounds that failed to meet structural or chemical requirements necessary for molecular docking, such as having undefined valency, missing atoms, unresolved stereochemistry, or failure to generate a valid 3D conformation during minimization. Consequently, the remaining 60 well-prepared compounds were selected for molecular docking to the active binding site of IKKα (Supplementary data Table S2).

To provide a comparative docking performance of the screened compounds, three IKK known inhibitors, BMS-345541, IMD-2560, and TPCA-1, were included as reference compounds. BMS-345541 exhibited a CDocker energy of −35.3389 kcal/mol and the strongest interaction energy of −50.2900 kcal/mol, supporting its high interaction role but less overall docking score. IMD-2560 showed a moderate binding affinity to IKKα with a CDocker energy of −32.9077 kcal/mol and interaction energy of −34.2859 kcal/mol. TPCA-1, another IKK inhibitor, demonstrated the weakest binding with a CDocker energy of −29.1605 kcal/mol and interaction energy of −31.641 kcal/mol. These reference values provided a comparative framework, highlighting that several natural compounds identified displayed equal or superior binding characteristics.

**Figure 3:**
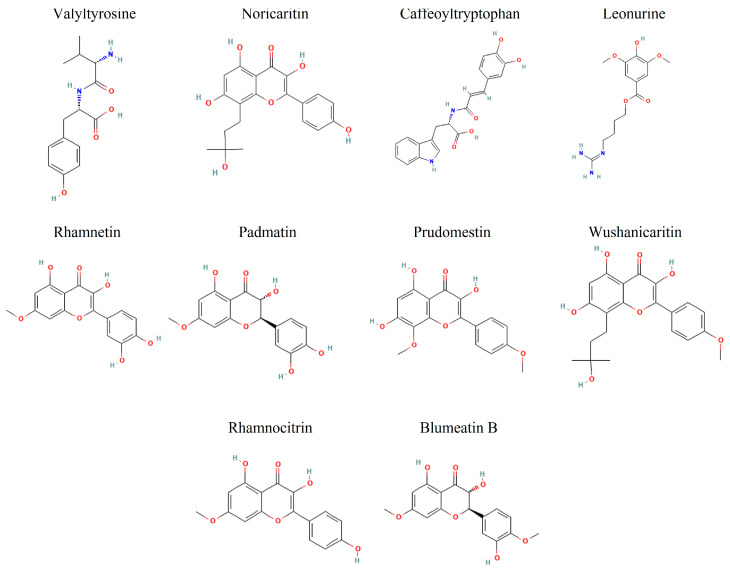
The 2D representation of the top 10 docked compounds against IKKα.

Among the top 10 screened compounds (Figure 3), Valyltyrosine emerged as the top-ranked compound with the most favorable CDocker energy (−47.7948 kcal/mol) and a strong interaction energy (−44.1606 kcal/mol), indicating a potentially stable binding configuration with the target. Noricaritin followed, exhibiting CDocker energy of −40.1424 kcal/mol and a stronger interaction energy of −46.5164 kcal/mol, suggesting its binding is more dependent on specific interactions. Caffeoyltryptophan, with CDocker energy of −38.5329 kcal/mol and interaction energy of −38.7883 kcal/mol, showed balanced binding properties (Table 1). Leonurine also displayed a relatively high CDocker energy (−35.8290 kcal/mol) but the lowest interaction energy (−48.1231 kcal/mol) among the group, implying robust intermolecular interactions despite its overall binding energy. Compounds like Rhamnetin and Padmatin showed comparable CDocker energies (−35.3055 and −33.0318 kcal/mol, respectively) but differing interaction energies (−40.3473 and −43.3144 kcal/mol), highlighting differences in their binding efficiency. Other compounds, including Prudomestin, Wushanicaritin, Rhamnocitrin, and Blumeatin B, displayed progressively less CDocker energies, yet their interaction energies suggested the presence of meaningful binding interactions, as summarized in Table 1.

Hypothetically, compounds with lower CDocker energy generally correlate with better stability within the binding pocket. However, interaction energy variations may indicate differences in the strength and nature of intermolecular forces, such as hydrogen bonding, hydrophobic interactions, or ionic contacts. Overall, Valyltyrosine, Noricaritin, Caffeoyltryptophan, Leonurine, and Rhamnetin manifested the most favorable docking profiles. Valyltyrosine is a dipeptide commonly found in fermented soy products and certain protein-rich plant sources. Noricaritin is a flavonoid derivative predominantly isolated from species within the Carthamus genus (e.g., Carthamus tinctorius, commonly known as safflower), which has been traditionally used in Chinese medicine. Caffeoyltryptophan is an esterified amino acid compound identified in several plant-based foods, especially those rich in hydroxycinnamic acids and indole-containing compounds, such as coffee beans and legumes. Leonurine is an alkaloid found in Leonurus japonicus (motherwort), a plant widely used in East Asian traditional medicine for its anti-inflammatory and cardioprotective properties. Rhamnetin, a naturally occurring flavonoid, is found in several medicinal plants, including Sophora japonica and Clove (*Syzygium aromaticum*), and has been studied for its antioxidant and anti−inflammatory effects. Moreover, Padmatin, Prudomestin, Prudomestin, Wushanicaritin, Rhamnocitrin, and Blumeatin B also showed the inhibitory potential against the target protein given their combination of strong CDocker and CDocker interaction energies. However, the real-time stability needs to be explored for these compounds in molecular dynamics simulations.

### 2.3. Molecular Docking Interactions

The molecular docking interaction analysis for the top 10 screened compounds was conducted to evaluate their interactions within the active site of the target protein. The results revealed that all compounds successfully docked within the active region and formed significant intermolecular interactions, including hydrogen bonds and salt bridges. While hydrophobic interactions were also observed (Supplementary data Figure S1), the primary focus was on stronger interactions that contribute significantly to binding stability, so only hydrogen bonds and salt bridges were discussed deeply. Valyltyrosine exhibited strong binding, forming one hydrogen bond and one salt bridge with bonding distances of 2.23 Å and 1.83 Å, respectively, suggesting highly stable interactions. Noricaritin, which ranked second in molecular docking energy after Valyltyrosine, demonstrated a notable interaction profile by forming three hydrogen bonds (one with Thr23 and two with Cys98) indicating robust anchoring within the active site. Caffeoyltryptophan displayed a complex interaction network, engaging with multiple amino acids, including Asp102, Cys98, Gly22, and Lys146. Its bonding distances ranged from 1.85 Å to 2.74 Å, highlighting its vast binding mechanism. Leonurine, ranked fourth, formed a single hydrogen bond with Arg20 at distances of 1.83 Å and 2.37 Å, implying a reliance on electrostatic stabilization. Similarly, compounds such as Rhamnetin, Padmatin, and Rhamnocitrin each formed two hydrogen bonds, targeting key residues like Cys98 and Thr23, while maintaining moderate binding affinities (Table 2).

**Figure 4:**
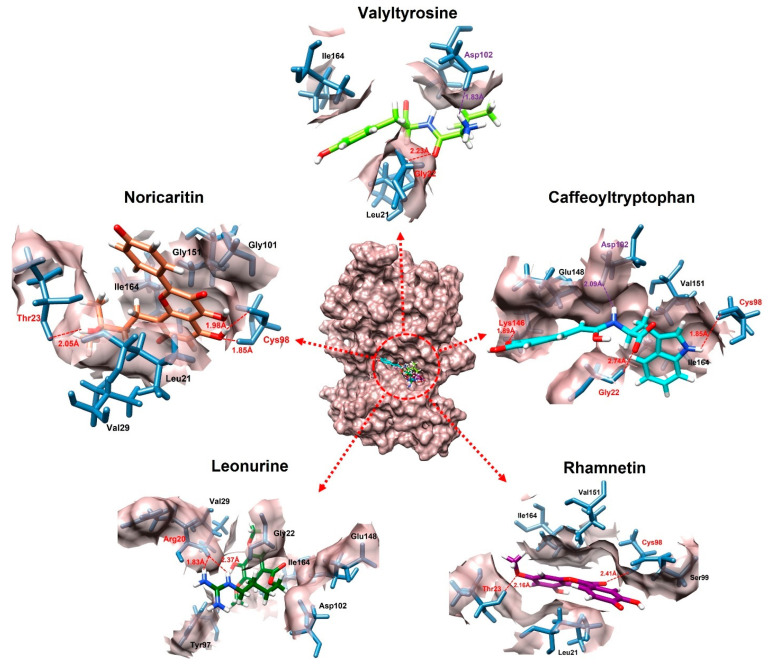
The interactions of the top five docked compounds against the active region of the IKKα. The salt bridges are colored as purple, hydrogen bonds are colored red, while the amino acids interacting hydrophobically are colored as black.

In contrast, Prudomestin, Wushanicaritin, and Blumeatin B formed only one hydrogen bond each, focusing primarily on key residues such as Cys98 and Glu148. Despite having fewer interactions, the precise targeting of critical residues by these compounds suggests their potential as inhibitors. For instance, Wushanicaritin’s interaction with Cys98 at 2.11 Å indicates a strong binding focus on this residue. Among the compounds docked, the graphical depiction of the interactions of the top five compounds is manifested in Figure 4; the remaining five compounds’ interactions are manifested in the Supplementary data Figure S2.

A graphical depiction of the interactions formed during the molecular docking analysis is represented below.

### 2.4. Molecular Dynamic Simulation

To further evaluate the stability of the top 10 screened compounds against IKKα in comparison with BMS-345541 as a reference, 100 ns molecular dynamics (MD) simulations were performed using GROMACS. BMS-345541 was chosen based on its high performance in molecular docking studies, while the MD results of the other two reference compounds have been provided in the Supplementary data Figure S3. This analysis provided a dynamic assessment to confirm the potential of these compounds as effective inhibitors of IKKα.

#### 2.4.1. Root Mean Square Deviation Analysis

**Figure 5:**
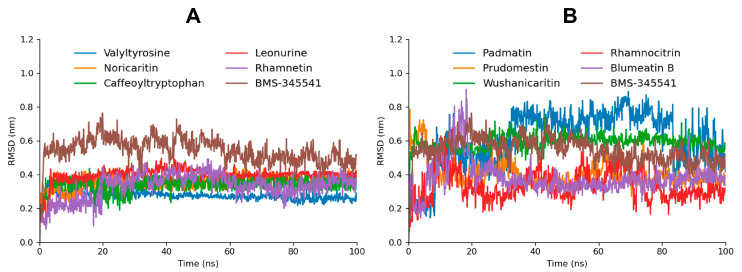
The RMSD graphs of the top five screened compounds compared to BMS-345541 are depicted in graph (**A**), while the RMSD graphs of the five following top docked compounds compared to BMS-345541 are depicted in graph (**B**).

A comprehensive RMSD (Root Mean Square Deviation) analysis was performed for the top 10 screened compounds to evaluate their real-time stability when complexed with the target protein. The RMSD results revealed that the top five screened compounds (Valyltyrosine, Noricaritin, Caffeoyltryptophan, Leonurine, and Rhamnetin) exhibited highly stable RMSD profiles throughout the molecular dynamics (MD) simulation as compared to BMS-345541, indicating consistent binding and minimal structural deviations (Figure 5). These findings align well with their superior docking scores, suggesting that their initial binding conformations remained stable throughout the simulation. Among the remaining five compounds, Wushanicaritin and Blumeatin B also displayed relatively stable RMSD values, albeit with slightly higher fluctuations compared to the top five compounds. Notably, Blumeatin B exhibited significant instability at the start of the MD simulation, as evidenced by a highly fluctuating RMSD profile during the initial phase. However, after 21 ns, the RMSD values began to stabilize, reflecting eventual equilibrium and suggesting that the compound underwent conformational adjustments before settling into a stable binding state.

In contrast, Padmatin, Rhamnocitrin, and Prudomestin demonstrated highly fluctuating RMSD profiles throughout the simulation, indicating weaker stability and significant structural deviations within the binding pocket. These fluctuations suggest that their initial docking poses were less optimal or that their interactions with the target protein were insufficient to maintain a stable complex. This instability is consistent with their comparatively lower docking scores, highlighting a correlation between binding energy and structural stability during MD simulations.

#### 2.4.2. Hydrogen Bonds

**Figure 6:**
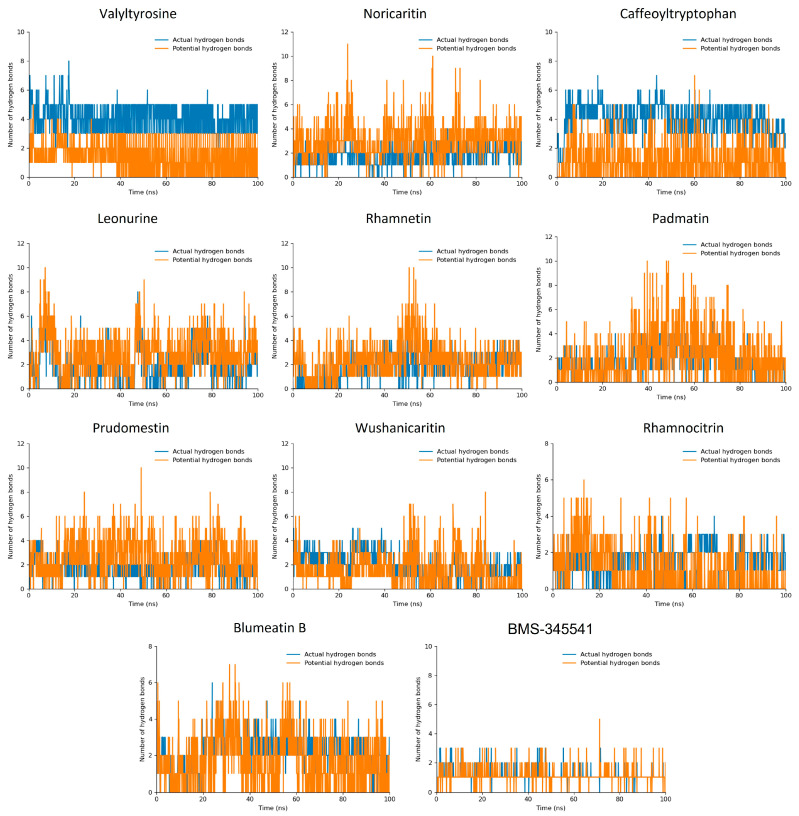
The hydrogen bond plots of the top 10 docked compounds. The blue bar lines highlight the actual hydrogen bonds while the orange bar lines highlight the potential hydrogen bonds.

The hydrogen bond plot analysis of the simulated compounds offered deeper insights into their binding interactions and stability during the 100 ns molecular dynamics (MD) simulation [46,47,48]. Among the screened compounds, Valyltyrosine and Caffeoyltryptophan demonstrated the most stable interaction profiles, forming two consistent actual hydrogen bonds throughout the simulation. Additionally, both compounds exhibited three potential hydrogen bonds, indicating a strong and stable binding affinity with the target protein. These findings align closely with their superior docking scores, further supporting their high binding efficacy and stable conformations under dynamic conditions. Noricaritin, Leonurine, and Rhamnetin, while showing slightly lower stability in their interaction profiles compared to Valyltyrosine and Caffeoyltryptophan, maintained an average of one consistent actual hydrogen bond throughout the simulation (Figure 6). Each of these compounds also exhibited a varying number of potential hydrogen bonds, suggesting intermittent but significant interactions within the binding pocket. Notably, Noricaritin’s interaction profile is consistent with its high docking score and RMSD stability, highlighting its robustness as a candidate. Leonurine and Rhamnetin, though slightly less stable, still demonstrated meaningful interactions that contribute to their moderate docking scores. BMS-345541, as the reference compound, manifested one interaction in most of the trajectories. Furthermore, the remaining compounds, including Wushanicaritin, Blumeatin B, Padmatin, Rhamnocitrin, and Prudomestin, displayed less consistent hydrogen bond profiles, averaging one or fewer actual hydrogen bonds during the simulation. While these compounds exhibited potential hydrogen bonds, the lack of stable interactions suggests weaker or less reliable binding. This observation aligns with their lower docking scores and fluctuating RMSD values, indicating that their binding modes may not be optimal for sustained interactions.

Comparatively, the stability and number of hydrogen bonds observed in Valyltyrosine and Caffeoyltryptophan underscore their strong potential as lead compounds, as their binding interactions were consistently maintained throughout the MD simulation. Noricaritin stands out among the other compounds for its stable hydrogen bonding and strong correlation with docking scores, making it a promising candidate for further study. In contrast, the weaker and less stable hydrogen bonding profiles of the lower-ranked compounds reinforce their reduced potential for further development. This analysis highlights the importance of stable hydrogen bonding in achieving high binding affinity and stability, providing critical insights into the molecular interactions that govern the efficacy of these compounds.

#### 2.4.3. MD Interaction Energy

**Figure 7:**
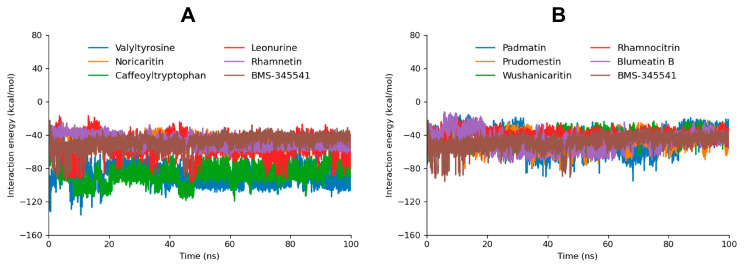
The interaction energy graphs of the top five screened compounds compared to BMS-345541 are depicted in graph (**A**). Moreover, the interaction energy graph of the five following compounds compared to BMS-345541 are depicted in graph (**B**).

The interaction energy analysis, including Coulomb (Coul-SR) and Lennard-Jones (LJ-SR) contributions [49,50], provides a detailed understanding of the binding energetics of the top 10 docked compounds with the target protein. The total interaction energy, derived from the sum of Coul-SR and LJ-SR energies, highlights the stability and strength of these interactions, supporting the findings from molecular docking and MD simulations (Figure 7). Valyltyrosine exhibited the most favorable total interaction energy (−89.16092 kcal/mol), with a significant contribution from Coul-SR energy (−67.9613 kcal/mol) and a moderate LJ-SR component (−21.1996 kcal/mol) as compared to the reference compound BMS-345541 (−48.7817 kcal/mol). These results reflect its strong electrostatic interactions and stable binding within the active site, consistent with its high docking score, stable RMSD profile, and the presence of stable hydrogen bonds during MD simulation. Similarly, Caffeoyltryptophan, with a total interaction energy of −87.0767 kcal/mol, showed a strong Coul-SR energy component (−61.8838 kcal/mol), aligning with its robust binding profile and consistent hydrogen bonding.

Noricaritin, Leonurine, and Rhamnetin exhibited moderate total interaction energies (−48.02299 kcal/mol, −55.6563 kcal/mol, and −48.6810 kcal/mol, respectively). The higher LJ-SR contribution in Noricaritin (−32.7132 kcal/mol) and Rhamnetin (−32.2729 kcal/mol) highlights their reliance on van der Waals interactions, which aligns with their moderate docking scores and stable but fewer hydrogen bonds. Leonurine, with a balanced contribution of Coul-SR (−25.9197 kcal/mol) and LJ-SR (−29.7366 kcal/mol) energies (Table 3), exhibited stable RMSD but fewer hydrogen bonds during the MD simulation, consistent with its intermediate interaction profile.

Compounds such as Padmatin, Prudomestin, and Wushanicaritin demonstrated slightly weaker total interaction energies (−45.3755 kcal/mol, −46.3691 kcal/mol, and −43.8694 kcal/mol, respectively). These results align with their fluctuating RMSD profiles and fewer stable hydrogen bonds observed during MD simulations, indicating moderate binding affinity. Blumeatin B, while having a lower total interaction energy (−47.1826 kcal/mol), showed a high LJ-SR contribution (−24.3362 kcal/mol) and displayed stabilizing behavior later in the MD simulation, suggesting potential for optimization.

Rhamnocitrin had the weakest total interaction energy (−39.7412 kcal/mol), driven by the lowest Coul-SR component among the compounds. This corresponds to its unstable RMSD profile and fewer hydrogen bonds, confirming its limited binding potential.

#### 2.4.4. MD Simulation Analysis

**Figure 8:**
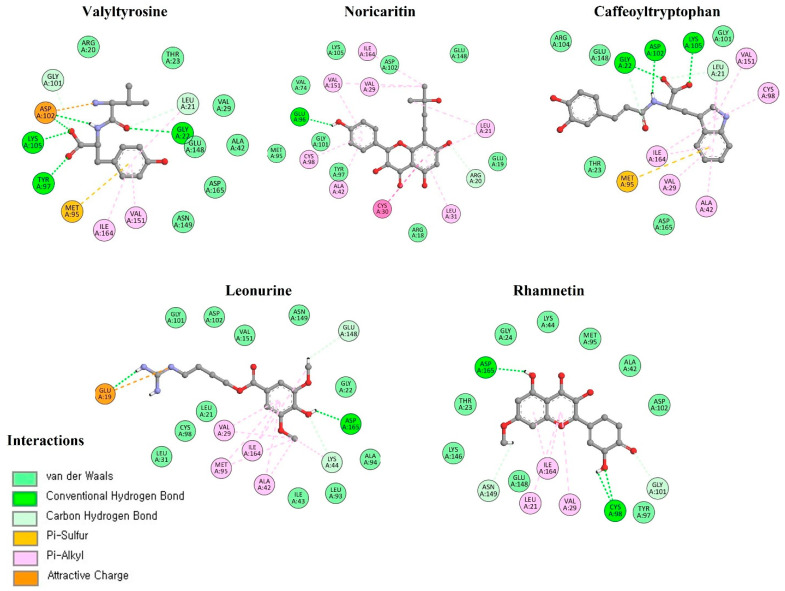
The interaction profile of top candidate compounds at 100 ns MD snapshot.

To evaluate the stability and binding persistence of the top five compounds within the active site of IKKα, snapshots were captured at the 100 ns mark from the molecular dynamics (MD) simulations for each protein-ligand complex. Detailed analysis of these structures revealed that all compounds consistently remained anchored within the active binding pocket throughout the simulation. The presence of stable hydrogen bonds and hydrophobic interactions further confirmed their compatibility and strong binding affinity toward IKKα (Figure 8).

Valyltyrosine and Caffeoyltryptophan consistently emerge as the top-performing compounds across all analyses. Their strong total interaction profile, stable RMSD graph, and robust hydrogen bonding patterns highlight their high binding affinity and dynamic stability, making them the most promising candidates for further exploration. Noricaritin, with a notable reliance on van der Waals interactions, exhibits moderate but consistent performance, aligning well with its intermediate docking score and hydrogen bond stability.

Compounds like Leonurine and Rhamnetin demonstrate a balanced interaction profile, stable RMSD profiles, and moderate hydrogen bonding, positioning them as viable secondary candidates. Overall, the interplay between the docking scores, RMSD stability, hydrogen bond analysis, and interaction profile highlights Valyltyrosine, Caffeoyltryptophan, and Noricaritin as top candidates for further development, while emphasizing the need to refine and optimize lower-ranked compounds.

### 2.5. Free Energy Calculation

The gmxMMPBSA analysis plays a critical role in molecular dynamics studies by providing a detailed estimation of binding free energies between a ligand and a target protein [51]. It allows the evaluation of the energetics of molecular interactions in a biologically relevant environment. This analysis is essential for understanding the thermodynamic stability of ligand–receptor complexes. By providing a more accurate prediction of binding affinities, gmxMMPBSA helps in identifying potent inhibitors and optimizing lead compounds.

**Figure 9:**
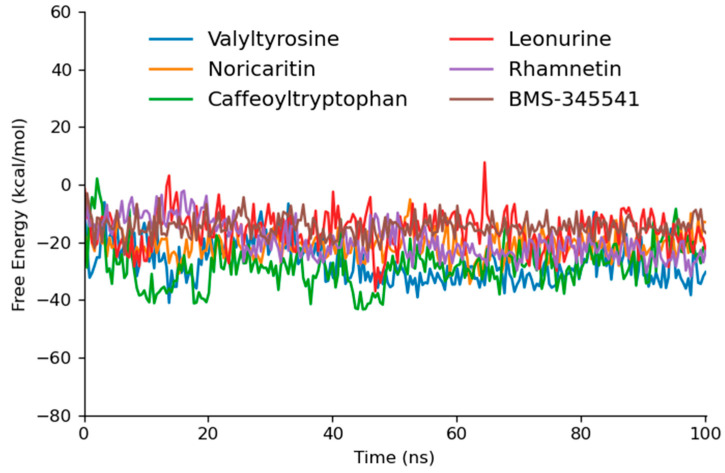
The gmxMMPBSA free energy graphs of the top five docked compounds that performed outstandingly at each step.

Caffeoyltryptophan exhibited the most favorable ΔG (−28.09 kcal/mol) among the tested and reference compounds, indicating its strong binding affinity and dynamic stability (Table 4). This result is consistent with its high interaction energy during MD simulations, where it demonstrated robust Coulombic and van der Waals interactions, as well as stable hydrogen bonding. Similarly, Valyltyrosine, with a ΔG of −27.07 kcal/mol, showed a comparable thermodynamic profile (Figure 9). Its excellent interaction energy in MD, coupled with consistent RMSD and hydrogen bond stability, underscores its potential as a lead compound.

Noricaritin, with a ΔG of −21.18 kcal/mol and the lowest standard deviation (4.50), exhibited a moderate binding affinity. Its lower thermodynamic stability compared to Caffeoyltryptophan and Valyltyrosine is mirrored in its interaction energy from MD simulations, which showed a higher reliance on van der Waals forces and less pronounced Coulombic interactions. This suggests that while Noricaritin is a strong candidate, its binding may rely on less stable interactions.

Leonurine and Rhamnetin demonstrated less favorable ΔG values of −15.74 kcal/mol and −19.20 kcal/mol, respectively. Both compounds, though displaying meaningful hydrogen bonding and van der Waals interactions, seem to lack the strong Coulombic contributions observed in higher-performing compounds, potentially limiting their overall binding strength

### 2.6. Experimental Validation

**Figure 10:**
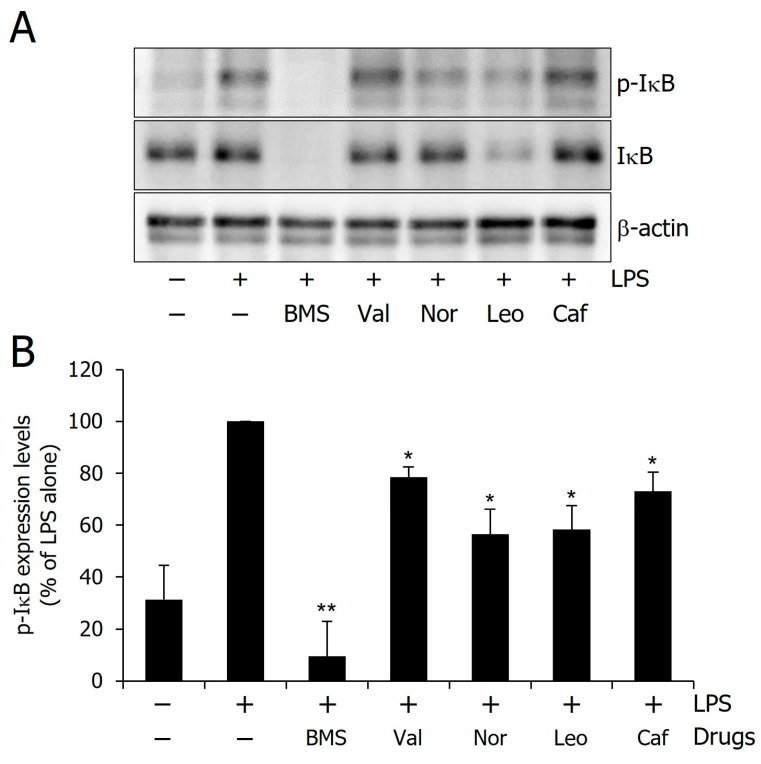
Inhibitory effect of the compounds on the IκBα phosphorylation. (**A**) Representative western blot image showing IκBα phosphorylation levels. (**B**) Quantitative analysis of phosphorylation levels. Single (*) and double (**) marks represent statistical significance in *p* < 0.05 and *p* < 0.01, respectively.

To validate the computational predictions that these compounds efficiently bind to IKKα, the phosphorylation levels of IκBα were examined in LPS-stimulated RAW 264.7 cells, as IKKα phosphorylates IκBα to initiate its proteasomal degradation [52,53]. A significant increase in IκBα phosphorylation was observed following the LPS treatment, while treatment with BMS-345541, a known IKKα inhibitor, led to a marked reduction in IκBα phosphorylation levels (Figure 10). The phosphorylation of IκBα was also attenuated by all tested compounds—Valyltyrosine, Noricaritin, Leonurine, and Caffeoyltryptophan—although their inhibitory effects were less pronounced than those of BMS-345541. Structural modifications may enhance their potency. Interestingly, despite the inhibition of IκBα degradation, a reduction in total IκBα levels was also observed. This effect may be attributed to the suppression of NF-κB-mediated transcriptional feedback, which normally restores IκBα levels [54,55,56,57]. These findings provide experimental validation of the computational predictions and suggest that these compounds could serve as potential IKKα inhibitors for further investigations.

A comparative analysis between in silico predictions and experimental outcomes underscores the complementarity of these approaches. Valyltyrosine and Caffeoyltryptophan, which showed the most favorable profiles in molecular docking, dynamics, and free energy calculations, also demonstrated notable inhibition of IκBα phosphorylation; however, the inhibition was less than expected, thereby validating their predicted binding affinity and stability. Interestingly, Noricaritin and Leonurine, which ranked slightly lower in computational analyses, displayed stronger biological activity than Valyltyrosine. This discrepancy suggests that certain dynamic or cellular factors may influence their in vitro performance and are not fully captured by computational models. These findings highlight the importance of combining in silico screening with biological validation to more accurately identify and prioritize lead compounds for further optimization.

## 3. Materials and Methods

### 3.1. Pharmacophore Modeling and Library Screening

The pharmacophore modeling was employed to generate key interaction features of receptor–ligand binding for the IKKα protein. The crystal structure of IKKα was retrieved from the Protein Data Bank (PDB ID: 5EBZ) to serve as the structural foundation for this analysis. Therefore, the protein kinase domain ranges from 15AA to 302AA was selected; the remaining amino acids were deleted from the data set for more focused analysis. Both the protein kinase domain of IKKα and its co-crystallized ligand were utilized to generate receptor–ligand interaction pharmacophore features using the RLIPG (Receptor–Ligand Interaction Pharmacophore Generation) module in Discovery Studio.

The Receptor–Ligand Interaction Pharmacophore Generation (RLIPG) protocol within Discovery Studio is an advanced tool for constructing pharmacophore models based on receptor–ligand interactions. This protocol offers several key advantages: it operates through a fully automated workflow, efficiently converting receptor–ligand complexes into detailed pharmacophore models; it allows for customizable constraints to precisely define interaction features between receptors and ligands; and it systematically generates all potential pharmacophore combinations, ranks them based on their selectivity scores, and prioritizes the top-ranked models for further investigation. These capabilities enable precise identification of the key molecular features driving selective binding at the IKKα active site.

### 3.2. Library Screening

To leverage this pharmacophore model, we utilized a curated natural product library for pharmacophore screening. The library, sourced from the MedChemExpress database (Natural Product Library Plus, catalog number HY–L021P, accessed on 10 October 2024), was selected for its diverse range of bioactive compounds such as 5540 natural products including Phenols (1383), Ketones, Aldehydes, Acids (930), Terpenoids (833), Alkaloids (622), Flavonoids (622), Phenylpropanoids (348), Steroids (277), Saccharides (179), Antibiotics (148), Quinones (100), Amino acids (78), Stilbenes (35), Cyclopeptides (8), Glucosinolates (7), and Others (961)

This library was screened against the pharmacophore model using Discovery Studio’s Library Screening tool, ensuring a robust and efficient identification of compounds that matched the pharmacophore features. This methodology enabled us to pinpoint compounds with a high likelihood of selectively interacting with the IKKα binding pocket, with the fit value ranging from 4.6486 to 1.2752 × 10^−6^. Out of 5540 compounds, only 82 compounds were screened according to six pharmacophore maximal features. The comprehensive screening process not only ensured high specificity but also facilitated the identification of diverse scaffolds that can be further optimized. By integrating pharmacophore generation and screening with a carefully selected compound library, this study provides a structured approach for the rational discovery of selective inhibitors targeting IKKα.

### 3.3. Molecular Docking

Molecular docking is a key computational technique used to predict the binding affinity and orientation of ligands within a target protein’s active site, based on calculated binding energies of protein−ligand complexes. In this study, the IKKα crystal structure (PDB ID: 5EBZ) was prepared by removing all co-crystallized ligands and water molecules, and hydrogen atoms were added using the receptor preparation tool in Discovery Studio. The CHARMm force field was employed for energy minimization and optimization of the receptor. Ligand structures were processed using the ligand preparation module, which involved generating relevant tautomers and stereoisomers, assigning ionization states at pH 7.4, correcting valence issues, and applying energy minimization using the CHARMm force field. Molecular docking simulations were performed using the CDocker module, a grid-based molecular dynamics (MD) simulated-annealing algorithm that uses CHARMm-based force field parameters to evaluate binding poses. A maximum of 10 poses per ligand was generated, and docking was carried out within a defined binding site radius of 12 Å around the co-crystallized ligand. The docking protocol incorporated default annealing dynamics settings and energy evaluation at the final minimization stage. The resulting protein–ligand complexes were ranked based on CDocker energy and CDocker interaction energy values (in kcal/mol), which represent the internal strain energy and binding strength of the ligand to the receptor, respectively, enabling accurate identification of the most favorable binding conformations.

### 3.4. Molecular Dynamics Simulations

The top 10 compounds with the lowest docking energies underwent a 100 ns molecular dynamics (MD) simulation to analyze their dynamic stability and interactions with the target protein. Simulations were set up using CHARMM36 force field parameters generated through the CHARMM-GUI server (https://www.charmm-gui.org/?doc=input/solution) (accessed on 2 October 2024), which also provided input files for GROMACS. The system was solvated in a cubic box with the TIP3P water model with a minimum distance of 10 Å between the protein surface and the box edge, neutralized with counter ions, and prepared with periodic boundary conditions.

The key interactions, including electrostatics and van der Waals forces, were calculated using the Verlet method with a 10 Å cutoff radius, while the particle mesh Ewald (PME) method ensured precise electrostatic calculations. Bond lengths were constrained using the LINCS algorithm. The systems underwent energy minimization via the steepest descent method and were equilibrated under NVT and NPT conditions before the production run.

Simulations were executed in GROMACS 2019.3 with a 2fs time step for accurate trajectory sampling. This workflow, supported by CHARMM-GUI scripted file for format conversion, enabled detailed structural and interaction analysis of the protein–ligand complexes, revealing critical insights into their dynamic stability and binding behavior.

### 3.5. Free Energy Calculation

The gmx_MMPBSA v1.6.3 tool is specifically designed to compute the end-state free energies of protein−ligand complexes using MD trajectory data generated in GROMACS. This tool employs the MM/PBSA (Mechanics/Poisson–Boltzmann Surface Area) method to estimate binding free energies by individually analyzing the protein–ligand complex, the isolated receptor, and the ligand within an explicit solvent environment. The binding free energy (ΔGbinding) of the lead compounds to the target protein was determined using the following equation:

ΔG_binding_ = G_complex_ − (G_protein_ + G_ligand_) (1)

In this equation, G_complex_ refers to the total energy of the protein–ligand complex, while G_protein_ and G_ligand_ represent the energies of the isolated protein and ligand in a solvated state, respectively.

### 3.6. Experimental Reagents and Cell Culture

Bacterial lipopolysaccharide (LPS) from Escherichia coli serotype 055:B5 was purchased from Sigma-Aldrich (St. Louis, MO, USA). BMS-345541, Valyltyrosine, Noricaritin, Leonurine, and Caffeoyltryptophan were purchased from MedChemExpress (Monmouth Junction, NJ, USA). RAW 264.7 macrophage cells were purchased from the Korea cell line bank (KCLB, cat #40071). RAW 264.7 macrophage cells were maintained in Dulbecco’s modified Eagle’s medium (DMEM; BYLABS, Luscience Corporation, Hanam-si, Gyeonggi-do, Republic of Korea) containing 10% heat-inactivated fetal bovine serum and 100 U/mL penicillin–streptomycin (Gibco, New York, NY, USA) at 37 °C, 5% CO_2_. Cells were incubated in 100 μM of various reagents for 1 h and then stimulated with 1 μg/mL of LPS for 4 h.

### 3.7. Western Blot Analysis

RAW 264.7 cells were pretreated with 100 µM BMS-345541, Valyltyrosine, Noricaritin, Leonurine, or Caffeoyltryptophan for 1 h prior to stimulation with 1 µg/mL LPS for 4 h. The cells were washed with PBS and lysed in PRO-PREP lysis buffer (iNtRON Biotechnology, Seongnam, Republic of Korea). Equal amounts of protein were separated on 10% SDS-polyacrylamide gel. The proteins were transferred to the Hypond PVDF membrane (Merck KGaA, Darmstadt, Germany) and blocked in 5% skim milk in TBST for 1 h at room temperature. Specific antibodies against p-IkB, IkB (1:1000; Cell signaling Technology, Danvers, MA, USA), β-actin (1:1000; Santa Cruz Biotechnology Inc., Dallas, TX, USA) were diluted in 5% skim milk. After thoroughly washing with TBST, horseradish peroxidase-conjugated secondary antibodies were applied. The blots were developed by the enhanced chemiluminescence detection (Abfrontier, GW Vieck, Seoul, Republic of Korea). Signals were captured and measured using the FUSION SOLO S imager (VILBER, Collegien, France).

### 3.8. Statistical Analysis

All values shown in the figures were expressed as the mean ± SD obtained from three independent experiments. The statistical significance was analyzed by a two-tailed Student’s *t*-test. Data with values of *p* < 0.05 were considered statistically significant. Single (*) and double (**) marks represent statistical significance in *p* < 0.05 and *p* < 0.01, respectively.

## 4. Conclusions

This study highlights the significance of selectively targeting IKKα as a therapeutic approach for cancers and inflammatory diseases driven by dysregulated NF-κB signaling. A rigorous computational workflow was employed to identify Valyltyrosine and Caffeoyltryptophan as the most promising candidates based on molecular docking, interaction energy, and free energy calculations. However, despite Valyltyrosine’s strong performance in computational analyses, its inhibitory effect on IκBα phosphorylation in experimental validation was less pronounced than expected. Interestingly, Noricaritin and Leonurine demonstrated notable experimental activity. This suggests that structural modifications to these compounds could further enhance their inhibitory potential. The experimental evaluation conducted in LPS-stimulated RAW 264.7 cells revealed that the selected compounds effectively attenuated IκBα phosphorylation. However, their inhibitory effects, while significant, were somewhat less pronounced than those of BMS-345541, indicating room for further optimization.

The selective inhibition of IKKα presents a significant therapeutic advantage, particularly in avoiding the adverse effects and toxicity associated with IKKβ inhibition, such as inflammatory skin diseases and increased epithelial vulnerability. By focusing on IKKα, this study enhances the understanding of its pivotal role in cancer progression and inflammation while identifying promising lead compounds for targeted therapeutic strategies. The integration of in silico and experimental approaches provides a strong foundation for further preclinical development, paving the way for more effective and safer IKKα-targeted therapies.

## Figures and Tables

**Table 1:** The docking energy values of the top 10 docked compounds, ranked considering the overall CDocker energy values.

Sr No	Compounds	CDocker Energy (kcal/mol)	CDocker Interaction Energy (kcal/mol)
1	Valyltyrosine	−47.7948	−44.1606
2	Noricaritin	−40.1424	−46.5164
3	Caffeoyltryptophan	−38.5329	−38.7883
4	Leonurine	−35.8290	−48.1231
5	BMS-345541 (Ref)	−35.3389	−50.2900
6	Rhamnetin	−35.3055	−40.3473
7	Padmatin	−33.0318	−43.3144
8	IMD-2560 (Ref)	−32.9077	−34.2859
9	Prudomestin	−32.3987	−42.4406
10	Wushanicaritin	−32.3714	−46.2998
11	Rhamnocitrin	−31.4653	−38.4445
12	Blumeatin B	−30.1299	−42.6128
13	TPCA-1 (Ref)	−29.1605	−31.641

**Table 2:** The hydrogen bonds and the salt bridges observed in the molecular docking interaction analysis; salt bridges are denoted with bold representation, whereas hydrogen bonds are depicted without emphasis.

Compounds	Interacting Residues	Binding Distances
Valyltyrosine	Gly22	2.23 Å
	**Asp102**	**1.83 Å**
Noricaritin	Thr23	2.05 Å
	Cys98	1.98 Å, 1.85 Å
Caffeoyltryptophan	**Asp102**	**2.09 Å**
	Cys98	1.85 Å
	Gly22	2.74 Å
	Lys146	1.89 Å
Leonurine	Arg20	1.83 Å, 2.37 Å
Rhamnetin	Cys98	2.41 Å
	Thr23	2.16 Å
Padmatin	Arg20	1.87 Å
	Glu148	1.97 Å
Prudomestin	Cys98	2.34 Å, 2.01 Å
Wushanicaritin	Cys98	2.11 Å
Rhamnocitrin	Thr23	2.19 Å
	Cys98	2.28 Å
Blumeatin B	Glu148	1.97 Å

The bolded amino acid residues represent the salt bridges and the length of the salt bridges.

**Table 3:** The MD interaction energy of the simulated compounds was calculated during the 100 ns MD simulation, representing both Coulombic and Lennard-Jones contributions.

Sr No	Compound	Interaction Energy (kcal/mol)		
		Coul-SR	LJ-SR	Total Energy
1	Valyltyrosine	−67.9613	−21.1996	−89.1609
2	Noricaritin	−15.3098	−32.7132	−48.0230
3	Caffeoyltryptophan	−61.8838	−25.1929	−87.0767
4	Leonurine	−25.9197	−29.7366	−55.6563
	BMS-345541	−22.4512	−26.3305	−48.7817
5	Rhamnetin	−16.4080	−32.2729	−48.6810
6	Padmatin	−20.3729	−25.0026	−45.3755
7	Prudomestin	−17.5392	−28.8298	−46.3691
8	Wushanicaritin	−13.8759	−29.9935	−43.8694
9	Rhamnocitrin	−11.7390	−28.0021	−39.7412
10	Blumeatin B	−22.8464	−24.3362	−47.1826

**Table 4:** The calculated gmxMMPBSA free energy of top six screened compounds.

Sr No	Compounds	ΔG_(TOTAL)_	Standard Deviation
1	Valyltyrosine	−27.07	6.94
2	Noricaritin	−21.18	4.50
3	Caffeoyltryptophan	−28.09	7.25
4	Leonurine	−15.74	6.38
5	Rhamnetin	−19.20	6.40
6	BMS-345541	−14.65	3.44

## Data Availability

The data that support the findings of this study are available from the corresponding author upon reasonable request.

## Supplementary Materials

The following supporting information can be downloaded at: https://www.mdpi.com/article/10.3390/molecules30092025/s1, Table S1: The predicted 10 pharmacophore hypotheses; Table S2: The molecular docking scores of all sixty compounds docked against IKKa; Figure S1: The 2D representation of the interactions of the top5 docked compounds; Figure S2: The 3D interactions images of five compounds following top five; Figure S3: The MD simulation results of the reference compounds IMD-2560 and TPCA1 against IKKα.
