# Supplementary material for: Investigating Natural Product Inhibitors of IKKα: Insights from Integrative In Silico and Experimental Validation
Source: Molecules. 2025 May 2;30(9):2025. doi: 10.3390/molecules30092025 (PMC12073143; doi:10.3390/molecules30092025)
Supplement: Supplementary file 1 [file molecules-30-02025-s001.zip › molecules-3417785-supplementary.pdf]

Investigating Natural Product Inhibitors of IKKα: Insights from Integrative *In Silico* Studies and Experimental Validation

Muhammad Yasir[1], Jinyoung Park[1], Eun-Taek Han[2], Jin-Hee Han[2],

Won Sun Park[3], Jongseon Choe[4], Wanjoo Chun[1,*]

[1]Department of Pharmacology, Kangwon National University School of Medicine, Chuncheon, 24341, Republic of Korea;

[2]Department of Medical Environmental Biology and Tropical Medicine, Kangwon National University School of Medicine, Chuncheon, 24341, Republic of Korea;

[3]Department of Physiology, Kangwon National University School of Medicine, Chuncheon, 24341, Republic of Korea;

[4]Department of Microbiology and Immunology, Kangwon National University School of Medicine, Chuncheon, 24341, Republic of Korea

Corresponding author: Dr. Wanjoo Chun, Department of Pharmacology Kangwon National University School of Medicine, Kangwon National University, Email: wchun@kangwon.ac.kr, Phone: +82-33-250-8853.

Table S1. The predicted 10 pharmacophore hypotheses.

Model	No. of Features	Features
Pharmacophore_01	6	AADDHHarom
Pharmacophore_02	5	AADDHarom
Pharmacophore_03	5	AADDH
Pharmacophore_04	5	ADDHHarom
Pharmacophore_05	5	ADDHHarom
Pharmacophore_06	5	AADHHarom
Pharmacophore_07	5	AADHHarom
Pharmacophore_08	4	AADD
Pharmacophore_09	4	ADDHarom
Pharmacophore_10	4	ADDH

Table S2. The molecular docking scores of all sixty compounds docked against IKKa.

Sr No	Compounds	CDocker energy	CDocker interaction energy
1	Valyltyrosine	-47.7948	-44.1606
2	Noricaritin	-40.1424	-46.5164
3	Caffeoyltryptophan	-38.5329	-38.7883
4	Leonurine	-35.8290	-48.1231
5	Rhamnetin	-35.3055	-40.3473
6	Padmatin	-33.0318	-43.3144
7	Prudomestin	-32.3987	-42.4406
8	Wushanicaritin	-32.3714	-46.2998
9	Rhamnocitrin	-31.4653	-38.4445
10	Blumeatin B	-30.1299	-42.6128
11	Physcion	-28.3728	-38.0264
12	Scutellarin methylester	-28.1752	-57.0025
13	Anhydroicaritin	-26.0809	-44.7249
14	Cyclo(Tyr-Leu)	-25.4734	-36.5509
15	Ombuin	-25.1073	-42.3096
16	Quercetagitrin	-24.5748	-50.8029
17	Kaempferol 7,4'-dimethyl ether	-22.7624	-37.4881
18	Quercetin 7,3',4'-trimethyl ether	-22.2705	-40.5268
19	Quercetin 7-glucuronide	-22.1898	-48.9978
20	Quercimeritrin	-18.9069	-48.3374
21	Rhodionin	-18.6609	-47.0697
22	Kaempferol-7-rhamnoside	-17.7399	-45.3768
23	kaempferol 7-O-glucoside	-16.6896	-52.9011
24	Vincetoxicoside B	-16.1731	-43.7902
25	Physcion 8-glucoside	-14.6496	-52.3941

26	Niazinin	-14.2198	-35.5476
27	Taxifolin 7-glucoside	-14.0170	-47.9515
28	Brassicin	-13.4878	-47.2608
29	Isoanhydroicaritin	-13.3634	-49.6227
30	Baicalin methyl ester	-12.5448	-40.2419
31	Emodin 6-O-|A-D-glucoside	-11.3192	-46.1565
32	2,3-Dehydrokievitone	-10.6217	-40.9456
33	Aloe Emodin 8-Glucoside	-9.42928	-47.1863
34	Moringin	-8.33579	-32.1155
35	Maohuoside A	-7.83442	-46.0784
36	Morusinol	-6.94902	-47.3385
37	Oleuroside	-6.4310	-48.8906
38	Taxifolin 7-Rhamnoside	-6.32572	-46.8856
39	Barbaloin A	-6.17477	-41.4743
40	Icaritin	-5.64424	-43.9385
41	Rhodiosin	-5.1747	-64.2357
42	Aloenin	-0.515353	-45.5088
43	Torachrysone 8-O-Glucoside	1.66603	-47.1086
44	Marmesinin	3.67699	-46.2372
45	Phytomelin	4.91932	-50.3486
46	Baohuoside I	6.13272	-60.7604
47	Biorobin	6.5782	-55.1818
48	Eriocitrin	6.76433	-45.9688
49	Icariside I	9.094	-51.6541
50	KushenolX	10.6267	-45.2536
51	Linocinamarin	11.4523	-55.9686
52	Lespenefril	12.5815	-51.6716
53	Chamaechromone	13.8068	-33.7075
54	Vitexin 4'-glucoside	13.9108	-51.0308
55	5-O-Methylvisammioside	14.5209	-53.2202
56	Protohypericin	25.9043	-38.3915
57	5'-Des-O-methylharringtonine	33.1023	-44.6955
58	Glucuronosylestradiol	33.2725	-32.7473
59	Cynanchagen+H31:K102in	50.0497	-44.0322
60	Rocaglamide	61.4353	-38.8128

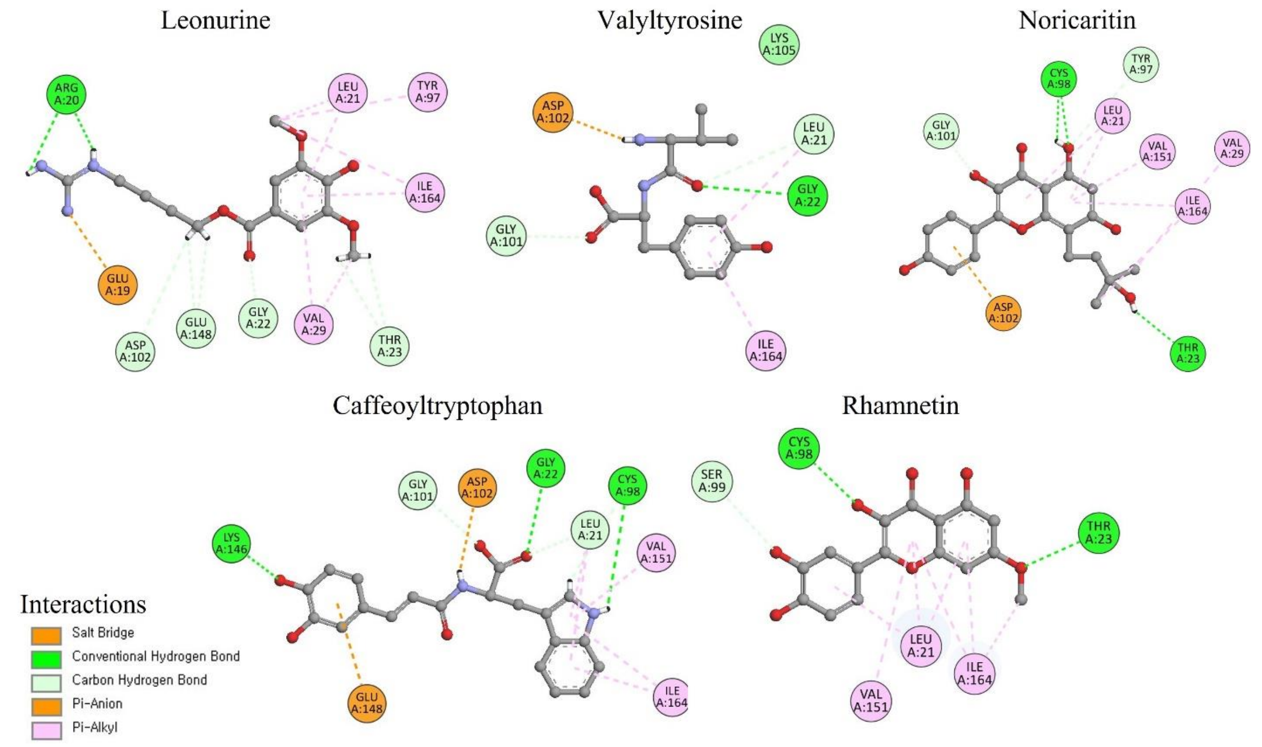

Figure S1. The 2D representation of the interactions of the top5 docked compounds

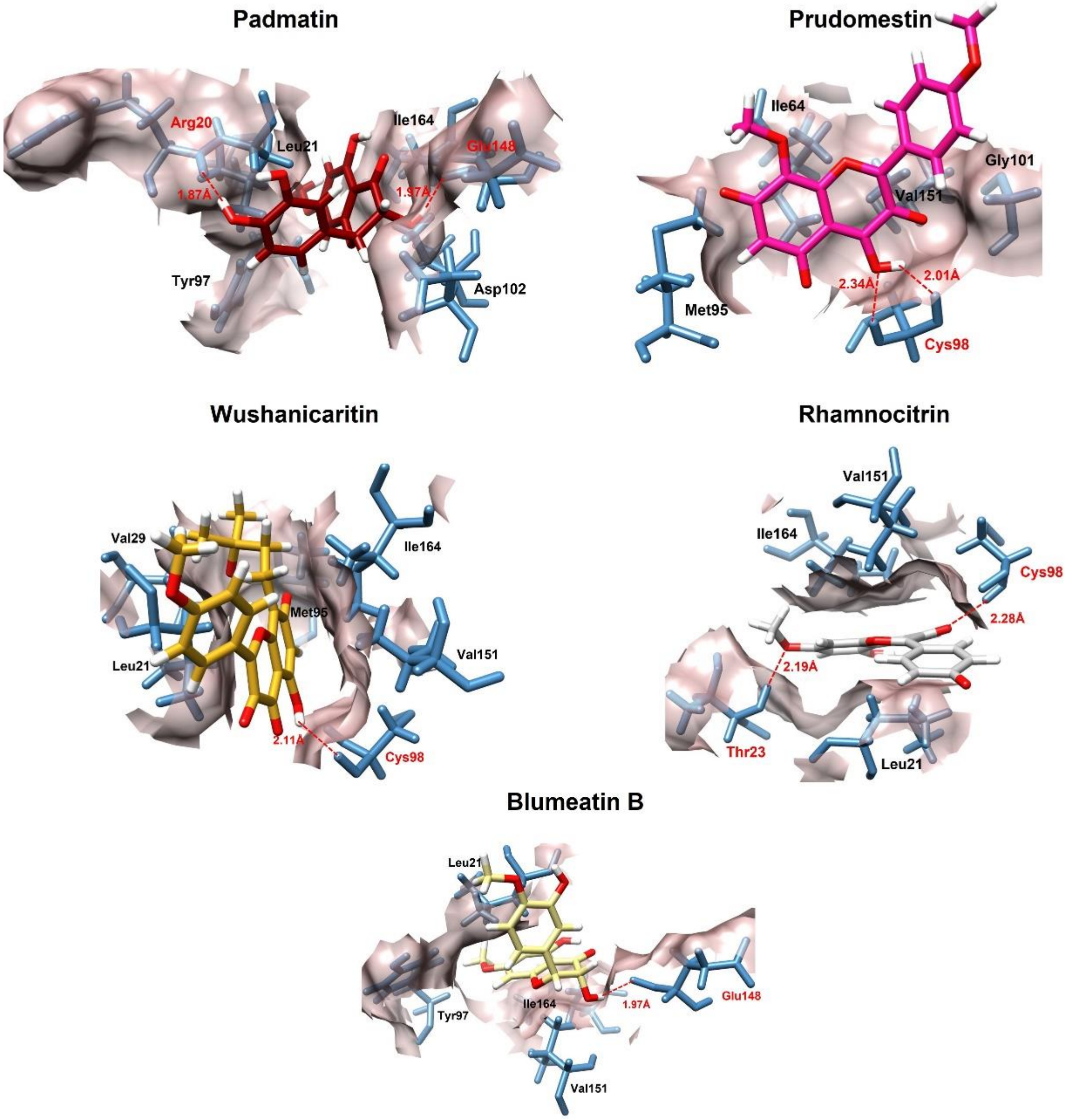

Figure S2. The 3D interactions images of five compounds following top five.

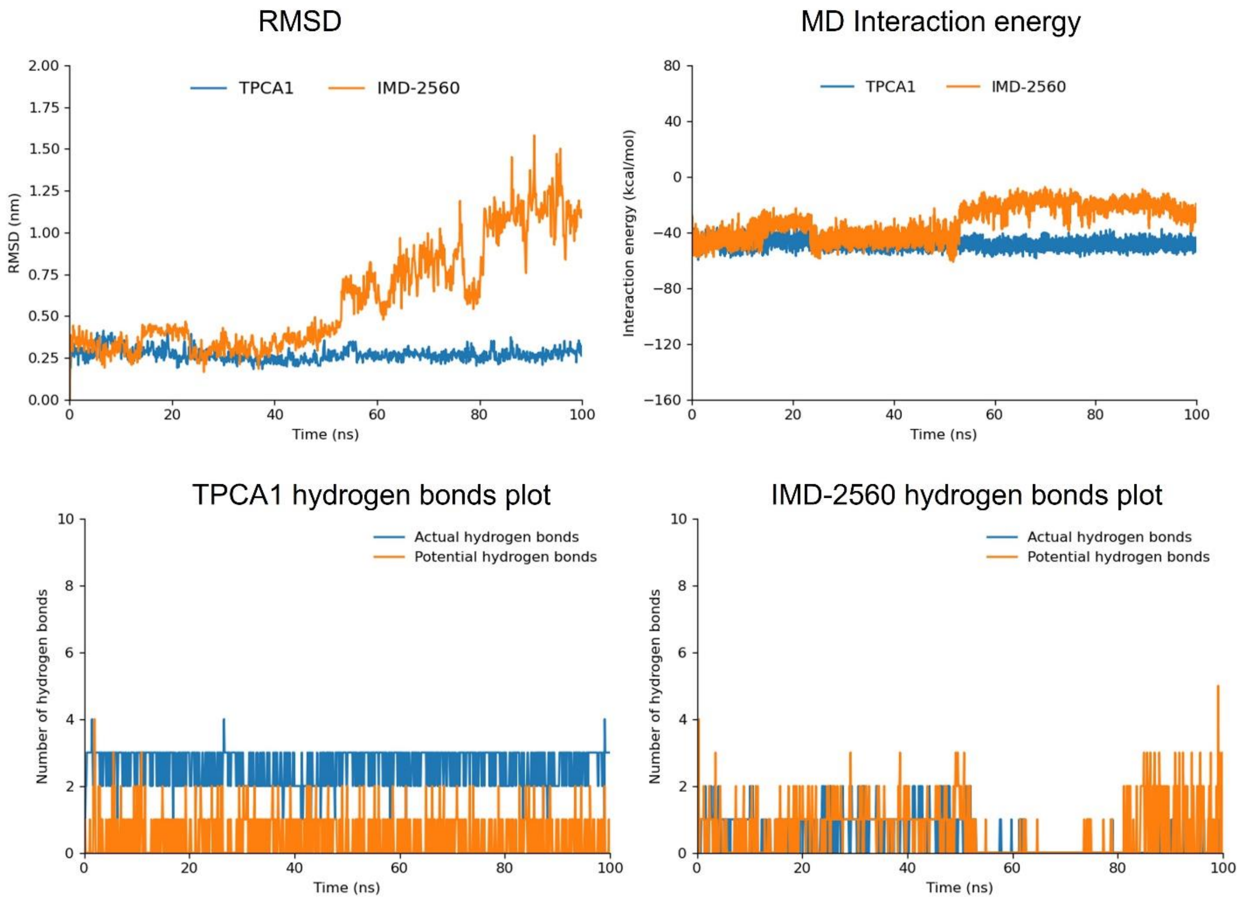

Figure S3. The MD simulation results of the reference compounds IMD-2560 and TPCA1 against IKKα.
